# An Interventional Study on Comprehensive Emergency Care and Trauma Registry for Road Traffic Injuries in India: A Protocol

**DOI:** 10.22114/ajem.v0i0.232

**Published:** 2019-08-05

**Authors:** Bontha V. Babu, Karthik Viswanathan, Aruna Ramesh, Amit Gupta, Sandeep Tiwari, Babu U. Palatty, Salish Varghese, Yogita Sharma

**Affiliations:** 1. Division of Socio-Behavioural & Health Systems Research, Indian Council of Medical Research, New Delhi, India.; 2. Department of Orthopaedics, PSMC-Shree Krishna Hospital, Anand, Gujarat, India.; 3. Department of Emergency Medicine, M.S. Ramaiah Medical College, Bengaluru, India.; 4. Division of Trauma Surgery and Critical Care, JPN Apex Trauma Centre, All India Institute of Medical Sciences, New Delhi, India.; 5. Department of General Surgery, King George’s Medical University, Lucknow, India.; 6. Department of Emergency Medicine, Jubilee Mission Medical College and Research Institute, Thrissur, India.

**Keywords:** Accidents, Traffic, Advanced Trauma Life Support Care, Emergency Medical Services, India

## Abstract

Road traffic injuries (RTIs) stands as one of the leading causes of mortality and morbidity across the globe. Effective injury surveillance systems and pre-hospital and in-hospital interventions set up in developing countries have shown promising results in controlling the problem. This study aimed to standardise and evaluate an evidence-based intervention for safety, efficacy and quality of post-crash pre-hospital and in-hospital trauma care services to improve the outcome in RTI victims. In addition, it establishes the android-based trauma registry for effective RTI surveillance.

This multi-centric, prospective, observational study is commissioned by the Indian Council of Medical Research (ICMR) as a National Task Force Project. This study is being conducted in five sites, viz., Anand, Bengaluru, Delhi, Lucknow and Thrissur located across India. Each centre will have a level I, two level II and three level III trauma hospitals. The study will be carried out in four phases namely: i) preparatory phase, ii) trauma registry establishment and pre-intervention data collection, iii) intervention and iv) impact evaluation. The preparatory phase, which lasts for four months includes the situational analysis pertaining to managing RTIs. Trauma registry will be initiated from the fifth month. Pre-intervention data will be collected for six months. The intervention will be conducted for six months in the form of prehospital notification, training for trauma care providers and trauma care quality improvement. Post-intervention data collection will continue for 12 months and the impact of the intervention will be assessed. The primary outcome measure will be early preventable mortality, defined as death at 24 hours after admission for patients with a calculated probability of survival >50% based on their injury severity score.

## Introduction

Road traffic injuries (RTIs) stands as one of the leading causes of mortality and morbidity across the globe. Approximately 1.24 million people die every year on the roads across the world, and another 20 to 50 million sustain non-fatal injuries as a result of RTIs. Eighty per cent of road traffic deaths occur in middle-income countries, which account for 72% of the world’s population but only with 52% of the world’s registered vehicles (1). The reasons for the high burden of RTIs in developing countries are - increasing in the number of motor vehicles, poor enforcement of traffic safety regulations, the inadequacy of health infrastructure and poor transport facilities (2).

In India, RTIs account for 15–50% of all injuries (3–5). Surveillance of RTIs is recommended to assess the burden, identify high-risk groups, establish an association with probable risk factors and plan interventions to control the occurrence and monitor impact (6). Effective RTI surveillance systems set up in developing countries have shown promising results in understanding the problem (7). It is the responsibility of the health system to ensure the establishment of necessary data systems including registries which will enable through the collection of information related to RTIs (8). However, systematic collection of road traffic data is not well developed in many developing countries including India and thus underreporting of injuries and deaths is common (9).

In the low and middle-income countries like India, injuries claim up to 18% deaths annually of which 50–60% of deaths occur either at the scene or en-route to the hospital (10). Faster response time and adequate prehospital care may dramatically improve these outcomes in critically injured patients (3). There is strong evidence to suggest that the establishment of an organized trauma system leads to improvement in patient outcomes, even in rural areas (4, 5). A recent prospective randomized study suggested that formal training in trauma care in the rural hospitals led to decreased length of time to transfer to appropriate hospitals as well as improved overall outcomes. It further demonstrated the cost-effectiveness and financial feasibility of such intervention in a rural setting (6). Timely and adequate care of a trauma patient is of utmost importance and it can dramatically impact the survival. There is a clear survival and functional benefit for critically injured patients to receive appropriate care within the first 60 minutes after injury. This concept is called the ‘Golden Hour’ (7, 11). The concepts of ‘pre-hospital care’ and ‘golden hour’ are still non-existent especially in the rural, remote and resource-poor regions (9). In this context, a study has been commissioned in five places in India by the Indian Council of Medical Research (ICMR) (12).

### Objectives of the study

The broad objective is to standardize structured evidence-based intervention for safety, efficacy and quality of post-crash pre-hospital and in-hospital trauma care services to improve the outcome in RTI victims. The specific objectives are (i) to study the prehospital trauma care system and emergency medical services (EMS) available in the district for identifying important components, which can improve the trauma health system in the district, (ii) to establish a prehospital care intervention through training of first responders and bystanders and sensitizing them about the importance of pre-hospital notification, (iii) to establish the intervention for emergency care by reorienting trauma care personnel and improving the facilities, (iv) to assess the overall impact of prehospital and in-hospital trauma care on the outcome of the RTI victims and (v) to establish a trauma registry system on electronic platform enabling data capturing through Android phones.

## Methods

### Study design and setting

This would be a multi-centric, prospective, observational, cohort study. The ICMR selected five Level I trauma centres (as per the classification given in WHO’s Guidelines for Essential Trauma Care (13)) from different parts of the country to form the national task force to conduct this study. These trauma centres are - Pramukhswami Medical College and Shree Krishna Hospital, Karamsad, Anand (West India), M.S Ramaiah Medical College and Hospital, Bengaluru (South India), Jai Prakash Narayan Apex Trauma Centre, All India Institute of Medical Sciences, New Delhi (North India), King George’s Medical University, Lucknow (North India) and Jubilee Mission Medical College & Research Institute, Thrissur (South India). In addition, two Level II and three Level III hospitals, meeting predefined requirements will be selected from each site. Thus, 30 hospitals (5 level I, 10 level II and 15 level III hospitals) will be involved in this study.

### Phases of the study

#### (I) Preparatory phase

**Administrative support**: Administrative support will be sought from the District Collector, the Superintendent of Police, the District Medical Officer, the Mayor and other functionaries in the city/district. The elected representatives of the city and district will be met to seek their support. A document highlighting the burden of RTIs will be prepared for each site and the same will be used for advocacy efforts.

**Identification and defining the role of partner hospitals (Level II and Level III hospitals)**: The partner hospitals would be identified on the basis of their geographical location and selected purposively at each of the five sites. Each level I hospital will select two level II and three level III hospitals. Once identified the roles and responsibilities of partner hospitals and the nodal officers at each hospital will be defined and given as written protocol.

**Situation analysis**: The situation of the district in terms of the epidemiology of RTIs and trauma care facilities available in the district would be done. The trauma care capacity will be assessed in all the 30 hospitals. Other facilities like EMS and ambulance services available will be listed in the documents. A situational analysis tool based on the WHO’s publication - “Guidelines for Essential Trauma Care” would be used for this purpose (13).

#### (II) Trauma registry establishment and pre-intervention data collection

Establishment of an online electronic registry for data collection would be completed by the end of 4 months of commencement of the project. The pre-intervention data will be collected through the registry in all participating centres. This phase will continue for 6 months. Trained research personnel would ensure data collection with a pre-set proforma in all participating hospitals.

**Trauma registry:** The registry will start functioning from the 5^th^ month of the project. After reviewing the pilot observations, an easy surveillance tool will be prepared for data collection in English, which might be translated for instruction in local languages for easy understanding by the research personnel. A detailed data set would be prepared to enter pre-hospital, in-hospital and post-discharge details of all the admitted patients.

**Data abstraction training:** Training for project personnel would be carried out at the institute of the principal investigator. Data collectors will be sitting in the emergency department of level 1 hospital for 24/7 on shift duties and record patient details into the prescribed registry proforma. In level II and level III centres, the emergency staff will collect data in the registry proforma and will hand over this proforma to the research team as per the agreement signed by these partner hospitals. These data will be transferred into the online registry.

The data management training including training on data dictionary (ICD, AIS), various types of data coding, injury severity scoring and training on other data management tools would also be undertaken for research personnel. This is aimed not only to help in the current project but also to help in capacity building for future trauma registries.

**Data collection:** Registry data collection will be done by research personnel under the supervision of the principal investigator and other senior staff based on the preset registry proforma. The proforma used for trauma registry is appended to this paper as Appendix.

#### (III) Intervention

The intervention phase will last for 6 months and shall include: (a) education of the first responders, (b) prehospital notification training, (c) acute trauma care training and improving the quality of care.

**(a) Education of the first responders:** The intervention in this study is to educate and train the first-responders like ambulance or emergency medical technician personnel, police and fire and safety services staff. The training will include basic education on the initial assessment of a trauma patient in the form of AIIMS Trauma First Responder (TFR) course. The training will be for 6–8 hours for one day conducted once every month for 6 months during the intervention period.

**(b) Prehospital notification training:** The recipient hospital needs to be notified about patient’s basic injuries and vitals while en-route, so that the receiving hospital has enough time to assemble a team, which is ready to receive and treat the patient upon arrival.

Level I trauma hospital will be issued mobile phones, with a dedicated toll-free number as part of the intervention. Panels comprising representatives of trauma hospital and feeding pre-hospital providers would be convened and, locally-tailored protocols and forms for the following made: (i) notification that specifies about whom, to whom and when the notification should be given, and what information should be communicated; (ii) responding at the receiving end, in particular assembling a receiving trauma team for cases requiring immediate resuscitation; (iii) documentation of call details, including the time of call, the information provided, and perceptions of its utility and any care that ensued; and (iv) structured handover upon arrival to the hospital.

**(c) Acute trauma care training and improving the quality of care:** AIIMS Trauma Assessment and Management (ATAM) course will be conducted providing training to the doctors and nurses catering to the RTI victims. ATAM would be held once every month at the level I hospital for a total duration of 6 months during the intervention phase. ATAM is a two-day course, which provides structured training in acute trauma management with special emphasis on skills training. Pre and post-test will be conducted with each course as a part of a quality improvement (QI) programme.

QI activities will be implemented according to the WHO guidelines to become focal points for training in their region. The intervention will include the WHO trauma QI training course for key clinicians and administrators, conducted during the first six months, followed by mentoring of development of the necessary structures and processes to conduct regular mortality and morbidity (M & M) meetings, preventable death reviews, audit filters, risk-adjusted benchmarking of key performance indicators where relevant and loop closure. The trainers and mentors will be drawn from the project leadership team.

#### (IV) Impact evaluation

Post-intervention trauma registry data collection would be carried out for 12 months after the intervention phase. Internal and external quality assurance would be carried out on a monthly basis. As mentioned at every stage of the study, the impact of the intervention will be measured through various outcome indicators, including the patients’ outcome like death, length of stay in hospital, morbidity, disability, etc. The necessary data for an impact evaluation will be obtained through the registry. Both pre- and post-intervention comparisons among various outcome variables will be made.

### Study outcomes

The primary outcome measure will be early preventable mortality, defined as death at 24 hours after admission for patients with a calculated probability of survival >50% based on their injury severity score. Secondary outcomes will be (i) the proportion of patients for whom the notification was indicated who had notification initiated, (ii) the proportion of patients who met criteria for assembly of a receiving trauma team for whom a team was assembled and (iii) ICU and hospital length of stay, etc.

### Data analysis

Quantitative data (continuous variables) will be presented as mean, standard deviation and range (if symmetrically distributed data) or as median and interquartile range (if asymmetrically distributed data). Ordinal and nominal data shall be presented as proportion and percentage. Student’s t-test will be used to compare the difference of means whereas; the Wilcoxon ranked sum test will be used to evaluate the statistical significance of the difference of medians. The Chi-square test or Fisher’s exact test shall be used to compare the difference of proportions (categorical variables). A p value of <0.05 was considered as the minimum level for statistical significance.

### Quality assurance

There will be internal and external quality monitoring at various levels. As an internal quality, a data entry specifies limits of variable value to avoid the entry-level errors. Hardcopy of the forms will be sent to the apex body on a weekly basis. Independent entry of the same forms will be carried out at apex centre, Delhi. The data manager will randomly select 10% the forms and cross-check with the data entry.

### Ethics

The study protocols were approved by the institutional ethics committees (IECs) of the respective level I hospitals/institutes. Each of the five IECs approved the study for the corresponding centre.

### Dissemination

The findings of the study will be shared with the pre-hospital and in-hospital personnel of the participating centres. The study findings will be disseminated through conducting workshops and publishing policy briefs. Dissemination workshops and policy briefs will primarily target policymakers, health administrators and other government functionaries of both state and national governments. Further, this research team will involve in advocacy campaigns to bring and implement trauma care guidelines in the country. The results will be presented at national and international scientific meetings and will be published in indexed scientific journals.

## Discussion

It is expected that this national task project shall study the existing pre-hospital and in-hospital services in geographically diverse districts from different regions of India, foster collaboration amongst researchers from different leading trauma centres of the country, facilitate capacity building in various districts and assess the effectiveness of interventional measures like pre-hospital notification and training of pre-hospital workers and in-hospital medical and paramedical personnel, etc. This study will provide comprehensive epidemiology and outcome of RTIs. Currently, in India, the EMS are fragmented and a few states have recently implemented a version of EMS that responds to a toll-free call for the medical emergencies (14). The findings will contribute to the setting of research and investment priorities to tackle the burden of RTIs. Also, evidence of the effectiveness of intervention through quality post-crash prehospital and in-hospital trauma care will emerge. This evidence is required for formulating trauma guidelines and policies.

This intervention research on safety, efficacy and quality of post-crash pre-hospital and in-hospital trauma care services to improve the outcome in RTI victims is timely. Majority of the RTI deaths is due to unreachability for basic and integrated emergency services, which lead to failure in utilizing golden hour. The intervention on prehospital care includes education and training for prehospital care and prehospital notification, standardized hospital selection, a mandatory checklist for persons attending RTI and prior notification of arrival. Pre-hospital notification alone has been found to be associated with reduced mortality and improved outcome (15). Faster response time and adequate prehospital care during this period can dramatically improve the outcomes in critically injured patients (16).

This study further aimed to educate and train the first-responders on components of prehospital notification including basic life support. The intervention will be made to ensure the availability of ambulances to transport the critically injured patients according to prior set criteria. Strengthening the quality of care within the study hospitals is another component intervention. It is evident that an organized trauma system leads to improvement in patient outcome (17). This kind of interventions is cost-effective and financially feasible to implement even in low resource settings (18).

The strengths of the study include implementation of trauma care intervention with trauma registry in electronic format across geographically diverse regions – some hospitals are completely urban whereas others are rural. The study also aimed to evaluate the effects of identical pre-hospital and in-hospital training among a large population of pre-hospital and in-hospital personnel. The analysis will have some limitations. We are not certain about the generalization of results. The selection of trauma care facilities is not random, rather they were selected purposively. However, these facilities have been selected from five different geographical zones to represent the country. Also, there is the limited external validity due to inclusion of level I trauma centre which is likely to receive complicated and severely injured patients and might show higher mortality rate, whereas the level II and III trauma centres might refer complicated and severely injured patients to higher centres and consequently a lower mortality rate might be observed in level II and level III hospitals. Also, we need to consider mitigating factors like introduction of government schemes for RTI patients (wherein even patients with minor injuries are likely to attend level-1 trauma centre) and re-configuration of medical and administrative responsibilities at trauma centres (wherein reduction of mortality rate might not be only due to prehospital notification or training of medical personnel). There are a lot of confounders in large multi-centric studies and reduction in mortality might not be dependent solely on the intervention components. Despite these limitations, the study followed rigorous methodology by adopting both national and international trauma care guidelines.

## Conclusions

Evidence of improvement of RTI victims through quality post-crash pre-hospital and in-hospital trauma care will emerge from this study. This study will further help to capture real-time data of road traffic injury patients attending the various hospitals. If the trauma training program for pre-hospital workers and in-hospital medical and paramedical workers is successful in improving the knowledge and various skill sets, then it could be implemented in different regions of India. The results of this study will contribute to the setting of research priorities and formulate trauma care guidelines and policies.
